# Naming racism in the public health classroom

**DOI:** 10.1371/journal.pone.0243560

**Published:** 2020-12-09

**Authors:** Nadia N. Abuelezam, Andrés Castro Samayoa, Alana Dinelli, Brenna Fitzgerald

**Affiliations:** 1 Boston College William F. Connell School of Nursing, Chestnut Hill, MA, United States of America; 2 Boston College Lynch School of Education and Human Development, Chestnut Hill, MA, United States of America; CUNY School of Medicine, City College of New York, UNITED STATES

## Abstract

**Objective:**

The discussion of racism within undergraduate public health classrooms can be highly influenced by local and national conversations about race. We explored the impact of local and national events on students’ ability to name racism on a public health exam highlighting the impact of racism on maternal and infant health disparities for Black mothers.

**Methods:**

We undertook this research within the context of an undergraduate introductory public health course at a primarily white institution in the Northeastern part of the United States. A qualitative content analysis of undergraduate student responses to a final exam question soliciting the importance of racism to health outcomes among Black mothers in the United States was undertaken. ANOVA tests were run to assess differences on naming racism, using semantic alternatives, and providing alternative explanations during three main time periods: prior to the election of the 45th president of the United States (pre-Trump), after the election (post-Trump), and after a nationally recognized racist campus incident.

**Results:**

Between the pre- and post-Trump periods we see no differences in naming racism or providing alternative explanations. We do see a reduction in the proportion of students providing semantic alternatives for racism in the post-Trump period (32.2 vs. 25.2%, p = 0.034). After the racist campus incident, we see increases in the proportion of students naming race (53.6 vs. 73.8%, p = 0.021) and decreases in the proportion providing an alternative explanation (43.1 vs. 12.9%, p = 0.004), but no differences in the proportion of students who used semantic alternatives.

**Discussion:**

This work lends itself to our understanding of how local climate affects public health teaching and may also influence students’ learning about important social and structural determinants of health. National and local climate should frame and guide public health teaching.

## Introduction

The field of public health prides itself on its ability to attend to the social and structural causes of poor health in vulnerable populations [1, 2]. Most recently, public health scholarship has sought to address social and structural determinants of health [3–6], which offer an opportunity to integrate emergent knowledge in the discipline with students’ engagement with scholarship on racism in their public health coursework [7–10]. Racism, understood as an ideological structure that “provides the rationalizations for social, political, and economic interactions between the races,” [11] is a fundamental cause of health [12, 13] and has been shown to have strong and lasting impacts on the health of minoritized populations [3, 14, 15]. Racism, a topic that is quite difficult to discuss within an educational context and may require specialized teacher training and education, has gained traction as a topic of increased research and discussion within public health scholarship and teaching [16–19]. While there is growing interest in advancing medical school curriculum to incorporate discussions around race and racism [20, 21], early exposure for undergraduate students aiming to continue in the basic sciences or medicine is important to countering the deceptive and insidious belief that race is genetic or biological [22, 23].

An important outcome of public health coursework in racism is increasing students’ abilities to name race and racism in their writing and verbal interactions. There is reason to believe that students’ comfort with discussing and writing about these issues may be highly influenced by the societal context in which they live [9, 24]. Just as we expect our public health students to better understand the context in which health is occurring, it is important to consider the sociopolitical and campus climate surrounding students’ learning of this complex material. We therefore recognize that events happening in a national and local context may, in fact, influence students’ learning and comprehension of course material.

For college students, particularly those in residential campuses, their college grounds can become microcosms of society with broader social patterns mediating their interactions [25]. At primarily White institutions (PWI) these microcosms, especially for students of color, can often reflect intensified racism and stigma at multiple points throughout their college education [26]. Despite efforts to increase the representation of students of color at PWIs [27], students of color remain a minority on these campuses, leading to heightened experiences of microaggressions, feelings of not belonging, and a lack of representation in course material and instructional staff [28]. On many campuses of PWI, large scale racist events have led to student organizing and protests mobilizing for increased engagement by campus administrators to address issues of racism, with a specific interest in how faculty address these dynamics in their instruction [29]. The movement to increase our understanding of people of color’s experiences within socially hostile environments and their resultant disparities within health is one way that students of color may better relate to material within public health classrooms [30, 31]. Undergraduate students who come into contact with public health classes may feel that these classes attend to issues and address societal and structural issues in a way that other classes may not [32, 33]. In particular, undergraduate public health classes that have a dedicated focus on addressing issues among vulnerable populations may provide an avenue for students of color and underrepresented students to engage with scholarship that is responsive to increased interests for greater representation and visibility in the classroom [34, 35].

Students seldom encounter examples that show the direct impact of racism on health. The field of maternal and child health offers a compelling example of well-documented evidence demonstrating the role that racism plays on health outcomes [36, 37]. Specifically, public health scholarship has exhaustively documented the elevated risk of maternal and infant complications during pregnancy for Black women (when compared to White women) [38–40].

This evidence is especially timely given the increased attention paid to the increasing maternal mortality rate for Black mothers in the United States in recent years, largely due to public figures like Serena Williams, speaking out about their own maternal health experiences [41]. Ultimately, researchers have shown that the disparity in maternal and infant health outcomes among Black and White Americans is due to the effect of prolonged racism on Black mothers’ bodies [36, 37, 42].

Instructors in an introductory public health class used the example of differential maternal health outcomes to highlight how racism directly affects health. Instructors in this class then aimed to test students’ comprehension of this material by asking students to discuss the impact of racism on Black mothers’ health in a final exam question. Investigators analyzed how students demonstrated their comprehension of racism within their stated responses in the final exam question through qualitative coding and quantitative comparisons. By examining students’ responses over multiple semesters, investigators hope to examine the ecological impact of large societal changes (such as a presidential election) and local school-wide events (such as racist hate crimes taking place on-campus) on how students chose to respond to an exam question on racism, with specific attention to the semantic construction of students’ responses. The goal of the pedagogy in the course was to help students name racism as a fundamental determinant of health and health disparities. In effect, the study’s conceptual framework builds upon past sociological work evidencing individuals’ longstanding aversion to explicitly discussing racism [11]. The study design offers a nexus between scholarship on social justice, health, and educational research to refine current understandings of students’ use of the term racism and how this has changed over time and in response to events happening within society.

## Methods

### Ethical review

This study was reviewed and deemed exempt by the Boston College Institutional Review Board.

### Class/Classroom

The introductory public health class is offered at a PWI institution without a larger school of public health. The course gives a broad overview of the basic principles and tenets of public health and then explores applications of these tenets by focusing on different disciplines and applications. Some of these applications include: non-communicable diseases, communicable diseases, environmental health, and maternal and child health. Students who take the course are enrolled in a wide variety of majors and minors on campus including biology, psychology, nursing, and international studies. Students can take the course at any point during their academic career, and students from a wide range of academic years enroll in the class. The course counts towards the social science requirements of the liberal arts education. The course has been taught by a public health teaching team; each semester the composition of instructors varies. The same instructor (Author 1) delivered material on racism for the Spring 2015, Fall 2017, Spring 2018, and Fall 2018 semesters. Two different instructors delivered material for Spring of 2016 and Spring of 2017.

### Course material on racism

During the maternal and child health week of the course, instructors focus their discussion on the determinants of the disparity in preterm birth incidence and maternal mortality among Black and White mothers. A segment of the documentary “Unnatural Causes” entitled “When the Bough Breaks” is shown in class. The segment first aired on PBS in 2008 and was produced by Tracy Heather Strain, Randall MacLowry, and Eric Stange. This segment focuses on the disparities in maternal and infant health outcomes between Black and White mothers in the United States and provides evidence that embodied racism contributes to this disparity. After a portion of the documentary is shown, students engage in an instructor-led discussion around the reasons for the disparity with an emphasis on racism as the primary determinant.

### Exam

As part of the course requirements, students must complete a final examination that covers course material. As part of the final examination (given on paper), students must complete a number of short answer responses to questions that cover the application portion of the course. On the final exam, students are asked to respond to the question: “In ‘When the Bough Breaks’ possible reasons for the black-white gap in infant mortality and birth weight are examined. What is the PRIMARY explanation for this gap?”

Exams were collected for 6 non-consecutive terms over 3 years. Students’ exams were then de-identified by the instructor, scanned, and responses to this particular question were transcribed by two of the authors (Author 3 and Author 4).

### Qualitative analysis

Parent codes were created prior to qualitative analysis based on prior literature and the goals of the analysis [43].

Three main codes were developed: racism, alternative explanation, and semantic alternative. If an exam response included the word “racism” and attributed the infant mortality gap to racism, the exam response was coded positively for racism. If the exam response included a different explanation for the mortality gap that did not involve racism (most commonly socioeconomic status, education, or nutrition) then the exam response was coded positively for alternative explanation. Alternative explanations were coded to account for the theory that individuals are comfortable discussing issues about racism with “anything but racism” [44–46]. Finally, if the exam response attempted to discuss racism explicitly without using the term but used words like “discrimination”, “prejudice”, etc., it was coded as a semantic alternative. Inclusion of the word “racial” without explicit use of the word “racism” was deemed a semantic alternative. Semantic alternatives were coded to account for the fact that the term racism often feels loaded and uncomfortable. Despite the fact that the alternatives are not sufficient, individuals think that they can “write around racism” which may reduce their discomfort [46].

Two of the authors (Author 3 and Author 4) were responsible for coding the exam responses and added sub-codes to address unanticipated patterns in the exams. Each coder was responsible for half of the exam coding. The coders then switched exams and checked each other’s work to ensure inter-coder reliability. During debrief meetings, all authors of the paper discussed the coding process and examined specific examples to clarify coding disagreements.

### Periods of interest

Because the campaign and election of Donald Trump for President of the United States was tinged with racist rhetoric and speech, and because the news media highlighted many of the racialized aspects of the presidential election, we believe it is important to consider this event as an influence on student responses on the exams. Further, the particular institution students attended in this study had a hate crime occur on campus that received local and national attention. An enrolled student defaced a residence hall with racial epithets specifically targeted at Black students in December of 2018. The student response to this hate crime was organized and resulted in a number of events on campus to raise students’ awareness of how students of color deal with daily incidents of racism. We define three periods of interest for our analysis. Semesters in which the final exam was taken prior to the election of Donald Trump in November of 2016 will be called “pre-Trump.” Semesters in which the final exam was taken after the election of Donald Trump in November of 2016 will be called “post-Trump.” Semesters in which the final exam was taken after the hate crime on campus will be called “post-campus event.”

### Student demographics

Due to the retrospective nature of the study and in order to protect the identity of the small number of students of color in the course, demographic information (gender and race distribution) and academic contextual information (class year and major) were collected on the aggregate for each semester. This data was extracted by the Office for Institutional Research, Planning, and Assessment based on the enrollment rosters for the courses. This data on gender and race is collected by self-report at the beginning of every academic year prior to the submission of the fall enrollment survey to the National Center for Education Statistics [47].

### Quantitative analysis

The quantitative analysis was used to analyze the results from the qualitative coding of exams. An ANOVA test was run to assess global differences by time period. ANOVA tests were chosen due to the nature of the data (overall proportion of exams with particular code) for each semester and the fact that time period served as an independent comparable group. Different ANOVA tests were run for each of the three main parent coding categories: racism, alternative explanation, and semantic alternative. Comparisons in the proportion of exams with each code were made between all three time periods, pre/post Trump time periods, and pre/post campus event time periods. All analyses were run in SAS 9.4.

## Results

### Sample description

We analyzed exams from 402 students over 6 semesters from Spring of 2015 through Fall of 2018. Across all semesters, students were predominantly female (72.5–94.0%) and white (57.6–63.9%) (Table 1). Class year composition changed over time with third and fourth year students highly represented in the Fall 2017 (67.8%) and Spring 2017 (75.8%) semesters.

**Table 1:** Characteristics of students by semester by demographic and academic factors.

Semester	Total # of students	Female, N (%)	Non-White, N (%)	Third and fourth year, N (%)
Spring 2015	80	58 (72.5)	34 (42.5)	41 (51.3)
Spring 2016	65	53 (81.5)	25 (37.9)	41 (63.1)
Fall 2017	59	50 (84.7)	22 (36.1)	40 (67.8)
Spring 2017	66	51 (77.3)	28 (42.4)	50 (75.8)
Spring 2018	65	56 (86.1)	26 (39.4)	31 (47.7)
Fall 2018	67	63 (94.0)	27 (39.1)	31 (46.3)

### Quantitative analysis

**Fig 1:**
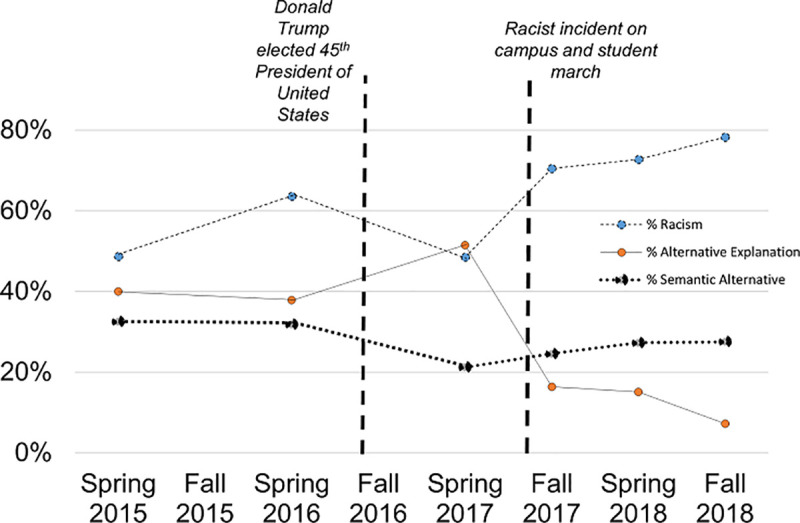
Coded proportions over time. Proportion of exam responses in each semester that named racism, provided an alternative explanation, or provided a semantic alternative to racism in each semester alongside national and campus events of interest.

A summary of the proportion of exam responses in each semester that named racism, provided an alternative explanation, or provided a semantic alternative to racism are outlined alongside national and campus events of interest in Fig 1.

When examining differences between the three time periods (pre-Trump, post-Trump, post-Campus event) we see no differences in the global ANOVA test for naming racism (F(2) = 6.89, p = 0.08, Table 2). We see a decrease in the proportion of students with an alternative explanation during the post-campus event period (12.9%, SD: 5.0%) when compared to the two prior time periods (F(2) = 42.3, p = 0.006). The proportion of students using a semantic alternative for racism varied across each time period with 32.2% (SD: 0.5%) in the pre-Trump period, 21.2% (SD: 0%) in the post-Trump period, and 26.5% (SD: 1.6%) in the post-Campus event period providing a semantic alternative (F(2) = 23.5, p = 0.015).

**Table 2:** ANOVA results examining the use of racism on undergraduate public health exams.

	All Events				Pre/Post Trump			Pre/Post Campus Event		
	pre-Trump	post-Trump	post-Campus event	global p-value	Pre-Trump	Post-Trump	global p-value	pre-Campus event	post-Campus event	global p-value
Racism	0.56 (0.11)	0.49 (0)	0.74 (0.04)	0.08	0.56 (0.11)	0.68 (0.13)	0.35	0.54 (0.09)	0.74 (0.04)	0.02
Alternative Explanation	0.39 (0.02)	0.52 (0)	0.13 (0.5)	0.006	0.39 (0.02)	0.23 (0.20)	0.33	0.43 (0.07)	0.13 (0.05)	0.004
Semantic Alternative	0.32 (0.01)	0.21 (0)	0.27 (0.02)	0.015	0.32 (0.01)	0.25 (0.03)	0.034	0.29 (0.06)	0.29 (0.02)	0.64

When examining differences between two time periods (pre/post-Trump) we see no differences in naming racism (F(1) = 1.1, p = 0.35) or providing alternative explanations (F(1) = 1.2, p = 0.33). We do see a reduction in the proportion of students providing semantic alternatives for racism (32.2 vs. 25.2%) in the post-Trump period (F(1) = 9.94, p = 0.034).

When examining the differences in the two time periods before and after the campus event, we see increases in the proportion of students naming racism (53.6 vs. 73.8%, F(1) = 13.5, p = 0.021) and decreases in the proportion providing an alternative explanation (43.1 vs. 12.9%, F(1) = 34.8, p = 0.004), but no differences in the proportion of students who used semantic alternatives (F(1) = 0.3, p = 0.62).

## Discussion

Overall, students’ ability to name racism as a reason for health disparities among Black and White mothers in the United States increased with time. Additionally, a large and racially charged national event, the election of the 45th President of the United States, seems not to have impacted students’ ability to name racism or provide alternative explanations. A local campus-level racist event did seem to be correlated with student responses naming racism and providing alternative explanations. Specifically, we see that students were more likely to name racism as a determinant after the racist event on campus and less likely to provide an alternative explanation. This work lends itself to our understanding of how local climate may impact public health teaching and may also impact students’ learning about important social and structural determinants of health.

There were significant differences in the proportion of students discussing racism before and after campus events. This result may suggest that students interrogated their own biases and had discussions given the proximity of the event to students’ everyday lives. Students cannot necessarily ignore racist incidents when they happen on campus, and this may therefore be a reason for the increased discussion of racism in these instances. Students’ perception and awareness of these issues may also be heightened and the events may cause these issues to be on the forefront of students’ minds. Given that the racial and ethnic compositions across students in different classes did not differ significantly, we argue that close proximity of racist incidents on college campuses correlates to students’ explicitly attributing racism as a health determinant that explains differential outcomes in maternal and child health between Black and white individuals. There is some evidence from psychological experiments with college students that hearing other students condone or condemn racism strongly influences a students’ verbalized beliefs [24]. It could, therefore, be true that because students were exposed to the topic and the use of the word outside of the classroom in social settings that these changes were occurring over time for this particular population.

Providing a semantic alternative to racism decreased in the post-Trump period suggesting, perhaps, that students were exposed to news articles and media around racism and it became a topic that was discussed more often and therefore used more often by students. A large proportion of students (20–40%) continue to use semantic alternatives to the word racism in their exams. These moments demonstrate the opportunity for public health educators to more clearly delineate the unequivocal evidence that demonstrates how racism is a structural determinant of health [13, 44]. This invitation for greater clarity in educators’ instructional framing of racism as a structural determinant of health aligns with current efforts for public health scholarship to explicitly name racism as a structural element explaining disparate health outcomes [2, 4]. Instructors in public health courses have an opportunity to deliver intentional instruction to discuss racism in the classroom and teach students about the importance of normalizing racism as a term used in public health scholarship. Given the stability of the proportion of students that engaged in semantic alternatives in their responses across various iterations of this course, we suggest that these semantic alternatives are emblematic of students’ inability to explicitly engage with racism as germane to discussions in public health.

Many of the students who take public health courses are pre-med students and are therefore used to taking classes in the basic sciences. When taking a public health course, they may find themselves in an unfamiliar learning environment given the explicit course materials’ focus on societal issues and issues related to race—especially on exams where answers can be open-ended written responses (unlike in in basic science exams). Because the majority of students in the classes each semester were biology or nursing majors, this may be one of the first opportunities for students to write on these topics [48]. Future studies should aim to examine differences by individual major declared. This could also lead to a better understanding of the role of the core curriculum and liberal arts education in educating students preparing for clinical practice to engage with issues related to social justice [49]. Future work should also examine the gendered nature of the discussion and the mediating role of students’ gender identity.

The conclusions of this analysis are limited by the study design. Specifically, the study was designed retrospectively and thus the information obtained regarding each student’s exam was limited. Additionally, final exams from the Fall of 2015 and the Fall of 2016 were not retained by instructors and were therefore not analyzed in this study. These semesters would have been important to our understanding of students’ reactions to national events. We were not able to pair personal demographic information to the individual responses which means that only ecological conclusions can be made from data analyses that exclude considerations of students as the unit of analysis. Additionally, the person instructing the class on maternal and infant health disparities changed each semester which suggests that there may be differences in teaching style or structure that may account for differences in students’ responses on the exam. Future work will aim to do a prospective study and will attain additional information on students’ demographic markers, thoughts, and opinions before and after the lecture to better understand students’ perspectives and learning regarding racism as a structural determinant of health.

While causal conclusions cannot be drawn from this ecological analysis, there are sufficient contributions within the analysis to inform future work. We were able to investigate how discussions of racism in a public health classroom influenced students’ writing about race. This type of work can help advance public health practice by ensuring that future public health students and leaders gain the skills they need to effectively communicate knowledge on structural and societal determinants of health. Additionally, this work suggests that administrators and faculty should be responsive in addressing local campus events that may lead to stigma on campus. Understanding public health has become more important in the context of the COVID-19 epidemic [50]. Additionally, heightened attention to structural racism and police violence have highlighted the importance of racism as a structural determinant of health. All students, regardless of their future careers, will benefit from accurate and evidence-based presentations of racism as a public health issue. Better understanding how to frame class material so that it is responsive to local and domestic environments may help produce more responsive public health professionals in the future.

## References

[1] Krieger N, Birn A-E. A vision of social justice as the foundation of public health: commemorating 150 years of the spirit of 1848. Am J Public Health. 1998;88(11):1603–6. 10.2105/ajph.88.11.1603 9807523 PMC1508556

[2] Bassett MT. # BlackLivesMatter—a challenge to the medical and public health communities. N Engl J Med. 2015;372(12):1085–7. 10.1056/NEJMp1500529 25692912

[3] Bailey ZD, Krieger N, Agénor M, Graves J, Linos N, Bassett MT. Structural racism and health inequities in the USA: evidence and interventions. The Lancet. 2017;389(10077):1453–63. 10.1016/S0140-6736(17)30569-X 28402827

[4] Gee GC, Ford CL. Structural racism and health inequities: old issues, new directions. Bois Rev Soc Sci Res Race. 2011;8(1):115–32. 10.1017/S1742058X11000130 25632292 PMC4306458

[5] Hardeman RR, Medina EM, Kozhimannil KB. Structural Racism and Supporting Black Lives—The Role of Health Professionals. N Engl J Med. 2016 12 1;375(22):2113–5. 10.1056/NEJMp1609535 27732126 PMC5588700

[6] Jee-Lyn García J, Sharif MZ. Black lives matter: a commentary on racism and public health. Am J Public Health. 2015;105(8):e27–30. 10.2105/AJPH.2015.302706 26066958 PMC4504294

[7] Njoku A. Teaching Health Disparities Awareness in Undergraduate Public Health Courses. Int J Scholarsh Teach Learn [Internet]. 2018 [cited 2019 Jul 8];12(2). Available from: https://eric.ed.gov/?id=EJ1186066

[8] Benabentos R, Ray P, Kumar D. Addressing Health Disparities in the Undergraduate Curriculum: An Approach to Develop a Knowledgeable Biomedical Workforce. CBE—Life Sci Educ. 2014 12 1;13(4):636–40. 10.1187/cbe.14-06-0101 25452486 PMC4255350

[9] Metzl JM, Petty J, Olowojoba OV. Using a structural competency framework to teach structural racism in pre-health education. Soc Sci Med. 2018;199:189–201. 10.1016/j.socscimed.2017.06.029 28689630

[10] Kernan WD, Basch CH. When the levees broke: A teaching tool to initiate discussions in undergraduate teaching of health disparities. Pedagogy Health Promot. 2017;3(1):50–5.

[11] Bonilla-Silva E. Rethinking racism: Toward a structural interpretation. Am Sociol Rev. 1997;465–80.

[12] Link BG, Phelan J. Social conditions as fundamental causes of disease. J Health Soc Behav. 1995;80–94. 7560851

[13] Phelan JC, Link BG. Is racism a fundamental cause of inequalities in health? Annu Rev Sociol. 2015;41:311–30.

[14] Krieger N. Does racism harm health? Did child abuse exist before 1962? On explicit questions, critical science, and current controversies: an ecosocial perspective. Am J Public Health. 2003;93(2):194–9. 10.2105/ajph.93.2.194 12554569 PMC1447716

[15] Castle B, Wendel M, Kerr J, Brooms D, Rollins A. Public health’s approach to systemic racism: A systematic literature review. J Racial Ethn Health Disparities. 2019;6(1):27–36. 10.1007/s40615-018-0494-x 29729001

[16] Hagopian A, West KM, Ornelas IJ, Hart AN, Hagedorn J, Spigner C. Adopting an Anti-Racism Public Health Curriculum Competency: The University of Washington Experience. Public Health Rep. 2018;133(4):507–13. 10.1177/0033354918774791 29847749 PMC6055294

[17] Metzl JM, Roberts DE. Structural competency meets structural racism: race, politics, and the structure of medical knowledge. AMA J Ethics. 2014;16(9):674–90. 10.1001/virtualmentor.2014.16.09.spec1-1409 25216304

[18] Came H, Griffith D. Tackling racism as a “wicked” public health problem: enabling allies in anti-racism praxis. Soc Sci Med. 2018;199:181–8. 10.1016/j.socscimed.2017.03.028 28342562

[19] Hardeman RR, Murphy KA, Karbeah J, Kozhimannil KB. Naming institutionalized racism in the public health literature: a systematic literature review. Public Health Rep. 2018;133(3):240–9. 10.1177/0033354918760574 29614234 PMC5958385

[20] White-Davis T, Edgoose J, Speights JB, Fraser K, Ring J, Guh J, et al Addressing racism in medical education an interactive training module. Fam Med. 2018;50(5):364–8. 10.22454/FamMed.2018.875510 29762795

[21] Hardeman RR, Burgess D, Murphy K, Satin DJ, Nielsen J, Potter TM, et al Developing a medical school curriculum on racism: Multidisciplinary, multiracial conversations informed by Public Health Critical Race Praxis (PHCRP). Ethn Dis. 2018;28(Suppl 1):271 10.18865/ed.28.S1.271 30116098 PMC6092164

[22] Nieblas-Bedolla E, Christophers B, Nkinsi NT, Schumann PD, Stein E. Changing How Race Is Portrayed in Medical Education: Recommendations From Medical Students. Acad Med J Assoc Am Med Coll. 2020; 10.1097/ACM.0000000000003496 32379145

[23] Tsai J, Ucik L, Baldwin N, Hasslinger C, George P. Race matters? Examining and rethinking race portrayal in preclinical medical education. Acad Med. 2016;91(7):916–20. 10.1097/ACM.0000000000001232 27166865

[24] Maudsley G, Strivens J. Promoting professional knowledge, experiential learning and critical thinking for medical students. Med Educ. 2000;34(7):535–44. 10.1046/j.1365-2923.2000.00632.x 10886636

[25] Lee A, Poch R, Shaw M, Williams R. Engaging Diversity in Undergraduate Classrooms: A Pedagogy for Developing Intercultural Competence: ASHE Higher Education Report, Volume 38, Number 2. John Wiley & Sons; 2012.

[26] Boyer PG, Davis DJ. Social Justice Issues and Racism in the College Classroom: Perspectives from Different Voices International Perspectives on Higher Education Research. Volume 8 Int Perspect High Educ Res 2013;

[27] Rendon L, Turner C, Garcia M, Nora A. Racial and ethnic diversity in higher education. New York NY Simon Schuster Cust Publ 1996;

[28] Museus SD. The culturally engaging campus environments (CECE) model: A new theory of success among racially diverse college student populations In: Higher education: Handbook of theory and research. Springer; 2014 p. 189–227.

[29] Griffin KA, Hart JL, Worthington RL, Belay K, Yeung JG. Race-Related Activism: How Do Higher Education Diversity Professionals Respond? Rev High Educ. 2019;43(2):667–96.

[30] Dundas KJ, Hansen V, Outram S, James EL. A “Light Bulb Moment” in Understanding Public Health for Undergraduate Students: Evaluation of the Experiential “This Is Public Health” Photo Essay Task. Front Public Health [Internet]. 2017 [cited 2019 Jun 27];5 Available from: https://www.frontiersin.org/articles/10.3389/fpubh.2017.00116/full 28589119 10.3389/fpubh.2017.00116 PMC5439459

[31] Curran N, Ned J, Winkleby M. Engaging Students in Community Health: A Public Health Advocacy Curriculum. Health Promot Pract. 2014 3 1;15(2):271–80. 10.1177/1524839913499349 23975798

[32] Albertine S. Undergraduate public health: preparing engaged citizens as future health professionals. Am J Prev Med. 2008;35(3):253–7. 10.1016/j.amepre.2008.06.005 18692738

[33] Wykoff R, Petersen D, Weist EM. On Academics: The Recommended Critical Component Elements of an Undergraduate Major in Public Health. Public Health Rep. 2013;128(5):421–4. 10.1177/003335491312800516 23997294 PMC3743296

[34] Smith SG, Nsiah-Kumi PA, Jones PR, Pamies RJ. Pipeline Programs in the Health Professions, Part 1: Preserving Diversity and Reducing Health Disparities. J Natl Med Assoc. 2009 9 1;101(9):836–51. 10.1016/s0027-9684(15)31030-0 19806840

[35] Robillard AG, Spencer SM, Richardson JB. Expanding the African-American studies paradigm to include health: A novel approach to promoting health equity. J Afr Am Stud. 2015;19(1):94–104.

[36] Krieger N. Epidemiology, racism, and health: the case of low birth weight. Epidemiology. 2000;11(3):237–9. 10.1097/00001648-200005000-00001 10784236

[37] Rich‐Edwards J, Krieger N, Majzoub J, Zierler S, Lieberman E, Gillman M. Maternal experiences of racism and violence as predictors of preterm birth: rationale and study design. Paediatr Perinat Epidemiol. 2001;15:124–35. 10.1046/j.1365-3016.2001.00013.x 11520405

[38] Loggins Clay S, Griffin M, Averhart W. Black/White disparities in pregnant women in the United States: An examination of risk factors associated with Black/White racial identity. Health Soc Care Community. 2018;26(5):654–63. 10.1111/hsc.12565 29488271

[39] Petersen EE, Davis NL, Goodman D, Cox S, Syverson C, Seed K, et al Racial/ethnic disparities in pregnancy-related deaths—United States, 2007–2016. Morb Mortal Wkly Rep. 2019;68(35):762 10.15585/mmwr.mm6835a3 31487273 PMC6730892

[40] Creanga AA. Maternal mortality in the United States: a review of contemporary data and their limitations. Clin Obstet Gynecol. 2018;61(2):296–306. 10.1097/GRF.0000000000000362 29561285

[41] Villarosa L. Why America’s black mothers and babies are in a life-or-death crisis. N Y Times. 2018;11.

[42] Liu SY, Fiorentini C, Bailey Z, Huynh M, McVeigh K, Kaplan D. Structural Racism and Severe Maternal Morbidity in New York State. Clin Med Insights Womens Health. 2019;12:1179562X19854778. 10.1177/1179562X19854778 PMC8842459 35237092

[43] Saldaña J. The coding manual for qualitative researchers. Sage; 2015.

[44] Blanchard FA, Crandall CS, Brigham JC, Vaughn LA. Condemning and condoning racism: A social context approach to interracial settings. J Appl Psychol. 1994;79(6):993–7.

[45] Bonilla-Silva E, Baiocchi G. Anything but racism: How sociologists limit the significance of racism. Race Soc. 2001;4(2):117–31.

[46] Harper SR. Race without racism: How higher education researchers minimize racist institutional norms. Rev High Educ. 2012;36(1):9–29.

[47] Ginder SA, Kelly-Reid JE, Mann FB. 2015–16 Integrated Postsecondary Education Data System (IPEDS): Methodology Report. NCES 2016–111 [Internet]. National Center for Education Statistics. National Center for Education Statistics; 2016 [cited 2020 Sep 15]. Available from: https://eric.ed.gov/?id=ED584137

[48] Williams DR, Mohammed SA. Racism and health I: Pathways and scientific evidence. Am Behav Sci. 2013;57(8):1152–73. 10.1177/0002764213487340 PMC3863357 24347666

[49] Williams DR, Lawrence JA, Davis BA. Racism and Health: Evidence and Needed Research. Annu Rev Public Health. 2019;40(1):105–25. 10.1146/annurev-publhealth-040218-043750 30601726 PMC6532402

[50] Abuelezam NN. Teaching Public Health Will Never Be the Same. 2020; 10.2105/AJPH.2020.305710 PMC7287535 32407134

